# Evaluation of the HIV-1 Rapid Recency Assay and Limiting Antigen Avidity Enzyme Immunoassay for HIV Infection Status Interpretation in Long-Term Diagnosed Individuals in Thailand

**DOI:** 10.3390/diagnostics15040444

**Published:** 2025-02-12

**Authors:** Supaporn Suparak, Petai Unpol, Kanokwan Ngueanchanthong, Siriphailin Jomjunyoung, Wipawee Thanyacharern, Sirilada Pimpa Chisholm, Nitis Smanthong, Thitipong Yingyong, Pilailuk Akkapaiboon Okada

**Affiliations:** 1 Department of Medical Sciences, Ministry of Public Health, Nonthaburi 11000, Thailand; petai.u@dmsc.mail.go.th (P.U.); kanokwan.n@dmsc.mail.go.th (K.N.); siriphailin.j@dmsc.mail.go.th (S.J.); wipawee.t@dmsc.mail.go.th (W.T.); sirilada.p@dmsc.mail.go.th (S.P.C.); s_nitis@kkumail.com (N.S.); pilailuk.o@dmsc.mail.go.th (P.A.O.); 2 Department of Disease Control, Ministry of Public Health, Nonthaburi 11000, Thailand; ying.thiti@gmail.com

**Keywords:** recent HIV infections, HIV-1 rapid recency assay, false recent rate, LAg-Avidity enzyme immunoassay, point-of-care testing, high-risk populations

## Abstract

**Background/Objectives:** Accurate surveillance of recent HIV infections is crucial for effective epidemic control and timely intervention. The Limited Antigen Avidity Enzyme Immunoassay (LAg-EIA) allows precise differentiation between recent and long-term HIV infections. To enhance accessibility, it has been developted into a point-of-care test, the Asanté™ HIV-1 Rapid Recency^®^ Assay (ARRA), a rapid immunoassay. This study evaluated the performance and false recent rates (FRRs) of the ARRA, interpreted both visually and via a strip reader, in comparison with the LAg-EIA. **Methods:** Plasma samples were collected from two groups: 634 long-term HIV-infected individuals, identified through routine diagnostic testing, who had not received antiretroviral therapy for over one year, and 224 individuals from high-risk populations. High-risk individuals, including pregnant women, female sex workers, and men who have sex with men, were selected based on behavioral and demographic risk factors. Concordance between the ARRA and LAg-EIA was assessed, and FRRs were calculated for both assays. McNemar’s test was used to evaluate agreement, while Spearman’s rho was applied to assess correlation between the two methods. **Results:** Visual interpretation of ARRA demonstrated perfect agreement with LAg-Avidity EIA results (FRR = 0.00%), while the strip reader misclassified two specimens as recent infections (FRR = 0.32%). McNemar’s test indicated no significant differences between the methods (*p* > 0.05). Moderate agreement (Spearman’s rho = 0.434) was observed between ARRA strip reader results and LAg-Avidity EIA optical density values. Among high-risk populations, ARRA misclassified one sample as recent, resulting in an inconsistency rate of 0.45%. **Conclusions:** This study highlights ARRA’s reliability in identifying long-term infections and its potential as a point-of-care tool. Its rapid results and ease of use make it a valuable asset for effective HIV surveillance, facilitating targeted epidemic monitoring and enhancing public health interventions.

## 1. Introduction

According to the UNAIDS 2024 Fact Sheet, there were approximately 39.9 million people globally living with HIV. In the same year, 1.3 million individuals became newly infected with HIV, and 630,000 people died from AIDS-related illnesses. Additionally, 30.7 million people were accessing antiretroviral therapy in 2023. In the Asia and the Pacific region, there were 6.7 million people living with HIV in 2023, with 300,000 new HIV infections and 160,000 AIDS-related deaths reported in the same year [1]. Thailand faces a significant HIV burden, particularly among key populations such as transgender women (29.7% prevalence) and men who have sex with men (16.7% prevalence). The urgency of addressing HIV transmission dynamics in these groups underscores the need for reliable and accessible diagnostic tools to support surveillance and targeted interventions [2,3,4].

To address these challenges, the Joint United Nations Programme on HIV/AIDS (UNAIDS) introduced the 95-95-95 targets, aiming to end the HIV epidemic by 2030. These targets focus on diagnosing 95% of all people living with HIV (PLHIV), ensuring 95% of those diagnosed receive ART, and achieving viral suppression in 95% of individuals on ART [5]. These statistics underscore the ongoing challenges in addressing the HIV epidemic globally and within specific regions, highlighting the need for continued efforts in prevention and treatment. A critical component in achieving these goals is the rapid identification of recent infections, which facilitates timely intervention and helps disrupt transmission chains.

Rapid tests for recent infections, also referred to as “recency assays,” have been developed as point-of-care (POC) tools to distinguish between recent and long-term infections. Among the widely used laboratory-based assays, the HIV-1 Limiting Antigen (LAg)-Avidity enzyme immunoassay (LAg-Avidity EIA) estimates antibody avidity to classify infections and has been instrumental in monitoring HIV incidence and transmission trends globally [6,7,8,9]. However, the LAg-Avidity EIA requires specialized laboratory infrastructure, highly trained personnel, and extended turnaround times, which limit its applicability in resource-limited settings and POC applications [10].

To overcome these limitations, rapid tests for recent infections enable HIV recency testing to be conducted within routine HIV programs in real time, significantly enhancing access to testing and generating actionable data for targeted prevention efforts [11]. The Asanté™ HIV-1 Rapid Recency^®^ Assay (ARRA) was specifically developed to address these challenges, offering rapid and reliable results at the POC. This lateral flow device provides visual or reader-assisted results within minutes, making it an accessible and timely tool for classifying recent and long-term infections. Studies conducted in sub-Saharan Africa, including Malawi and Rwanda, have demonstrated ARRA’s strong concordance with the LAg-Avidity EIA, underscoring its utility for public health surveillance and targeted interventions, particularly in high-risk populations [8,12].

This study evaluates the performance of ARRA and LAg-Avidity EIA in specimens from individuals with long-term HIV diagnoses in Thailand, comparing their interpretation outcomes to assess the feasibility of integrating rapid tests for recent infections into the national HIV surveillance framework. Additionally, the ARRA test kit was evaluated using samples from individuals with unknown infection periods, including pregnant women, female sex workers, and MSM. The inclusion of high-risk populations in HIV surveillance is critical for understanding transmission dynamics and tailoring public health interventions to prevent new infections. Real-time HIV surveillance using accessible and effective rapid tests for recent infections like ARRA could provide invaluable data to inform prevention strategies targeting these key populations.

By comparing ARRA outcomes with the established LAg-Avidity EIA, this study seeks to provide insights into ARRA’s utility as a POC tool in Thailand’s HIV surveillance system. The findings aim to contribute to the growing body of evidence supporting rapid tests for recent infections in resource-limited and high-prevalence settings, reinforcing their role in achieving UNAIDS 95-95-95 targets and ultimately ending the HIV epidemic in Thailand and beyond.

## 2. Materials and Methods

### 2.1. Ethical Review

This study was conducted in accordance with the ethical principles outlined in the Declaration of Helsinki. The study protocol received approval from the Department of Medical Sciences, Ministry of Public Health, Thailand (Approval No. 12/2565).

#### Data Transparency and Reproducibility

While privacy concerns preclude public access to raw data, aggregated results and detailed statistical methods have been thoroughly presented to maintain clarity and reproducibility. Anonymized datasets can be made available to qualified researchers upon reasonable request, subject to ethical approval and adherence to data-sharing agreements.

### 2.2. Specimens

The sample size of 634 for long-term infections represents the available specimens collected in 2013, prior to the implementation of the “Test and Treat” strategy in Thailand in 2014. At that time, ART eligibility was determined by treatment guidelines, which recommended treatment initiation only for individuals with CD4 cell counts below 350 cells/mm^3^ or those presenting with advanced HIV-related symptoms. All participants in this study had CD4 cell counts above this threshold and were therefore not eligible for ART under the 2013 guidelines. These specimens were evaluated to determine the false recent rate (FRR) using the Asanté™ HIV-1 Rapid Recency^®^ Assay (ARRA) (Sedia Biosciences Corporation, Beaverton, OR, USA) and the HIV-1 Limiting Antigen (LAg)-Avidity Enzyme Immunoassay (LAg-Avidity EIA) (Sedia Biosciences Corporation, Beaverton, OR, USA).

Additionally, the 224 specimens from high-risk groups were included to ensure representation of populations at heightened risk of HIV transmission and to examine ARRA’s performance across diverse demographics. These groups included pregnant women, female sex workers, and men who have sex with men (MSM), all of whom are at elevated risk for HIV infection. High-risk groups were classified based on epidemiological and behavioral criteria. Pregnant women were identified from antenatal care records. Female sex workers were classified based on self-reported occupation and engagement in transactional sex, and MSM were identified based on self-reported sexual behavior during pre-test counseling.

The inclusion of these distinct sample groups allowed for a comprehensive evaluation of the ARRA test kit’s diagnostic accuracy, with implications for its potential application in diverse HIV surveillance settings. By focusing on both long-term HIV infections and high-risk groups, the study provides valuable insights into the utility of ARRA as a point-of-care diagnostic tool for real-time HIV monitoring and targeted public health interventions.

### 2.3. Laboratory Methods

#### 2.3.1. HIV-1 Test Samples

Plasma specimens were tested following the Thai National Guidelines for HIV di-agnostics. Initial screening was performed using the Elecsys HIV Combi PT (Roche Diagnostics Ltd., Mannheim, Germany), which combines the detection of HIV antigens and antibodies. Reactive samples were further confirmed with the Serodia HIV-1/2 MIX (Fujirebio Inc., Tokyo, Japan), and the Alere HIV Combo (Alere Medical Co., Ltd., Chiba, Japan) to verify HIV seropositivity. All tests were conducted in accordance with the instructions specified in the respective package inserts.

Confirmed HIV-positive specimens were subsequently utilized for evaluating the Asanté™ HIV-1 Rapid Recency^®^ Assay (ARRA) and the HIV-1 Limiting Antigen (LAg)-Avidity Enzyme Immunoassay (LAg-Avidity EIA). These assays were used to interpret the infection status as recent or long-term among individuals diagnosed with HIV in Thailand.

The comprehensive testing workflow ensured high diagnostic accuracy and reliability, enabling a robust evaluation of the ARRA and LAg-Avidity EIA in the context of long-term HIV infections.

#### 2.3.2. Performance of HIV-1 Limiting Antigen (LAg)-Avidity Enzyme Immunoassay (LAg-Avidity EIA)

The HIV-1 LAg-Avidity EIA (Sedia Biosciences Corporation, Beaverton, OR, USA) was performed in accordance with the manufacturer’s protocol and previously established methodologies [4,9]. Reagents were equilibrated to room temperature, except for the TMB substrate, which was maintained at 25 °C, and goat anti-human IgG peroxidase, which was stored at −20 °C until use. Serum or plasma specimens were diluted with milk buffer, and 100 µL of each sample or control was added to individual wells of a microplate. Plates were incubated at 37 °C for 1 h, followed by four washes with wash buffer. Subsequently, 200 µL of dissociation buffer was added to each well, and the plates were incubated for 15 min at 37 °C. After another washing step, 100 µL of diluted goat anti-human IgG peroxidase was added and incubated for 30 min. Next, 100 µL of TMB substrate was added, and plates were incubated for 15 min. The reaction was terminated by the addition of 100 µL of 1 N H_2_SO_4_. Optical density (OD) at 450 nm was measured, and normalized OD (ODn) values were calculated using the formula:

ODn = Specimen OD/Median CAL OD

Specimens were tested in duplicate across two independent runs, and the median ODn values were used for analysis.

In the LAg-Avidity EIA, the screening run employed a normalized optical density (ODn) cutoff of ≤2.0 to classify a specimen as potentially recent, requiring confirmation through further testing. In the confirmatory run, a more stringent ODn cutoff of ≤1.5 was applied to definitively classify a specimen as recent.

This two-tier approach was designed to enhance the assay’s specificity by minimizing false positives while maintaining sensitivity during the initial screening. The lower threshold in the confirmatory run ensured greater accuracy in the final classification of recent infections, aligning with the assay’s validation studies and international recommendations for improving the precision of recency classification.

#### 2.3.3. Performance of Asanté™ HIV-1 Rapid Recency^®^ Assay (ARRA) Procedure

The Asanté™ HIV-1 Rapid Recency^®^ Assay (ARRA) (Sedia Biosciences Corporation, Beaverton, OR, USA) was performed according to the manufacturer’s protocol and methodologies outlined in previous studies [13,14].

For the procedure, serum or plasma specimens were first equilibrated to room temperature. A 5 µL sample was then collected using the collection loop provided in the kit, transferred into a buffer tube containing 0.5 mL of buffer, and mixed thoroughly. The test strip was then immersed into the buffer tube, incubated for 20 min, and removed. After incubation, the strip was dabbed on filter paper to remove any excess buffer before visual assessment. The results were subsequently read with the Asanté strip reader. To minimize bias, the visual results were recorded prior to the reader-based interpretation.

##### Interpretation of Results of ARRA

The interpretation of the Asanté™ HIV-1 Rapid Recency^®^ Assay (ARRA) results was based on both visual inspection and strip reader analysis.

##### Visual Interpretation

Long-term infection was identified by the presence of three distinct lines—a control line (CL), positive verification line (PVL), and long-term line (LTL). Recent infection was characterized by the appearance of only the control line (CL) and the positive verification line (PVL). For HIV-negative specimens, only the control line (CL) was visible, indicating no HIV infection.

##### Strip Reader Interpretation

PVL intensity: A measurement greater than 2.8 IU indicated an HIV-positive result, while a value below 2.8 IU indicated an HIV-negative result. LTL intensity: An intensity above 2.9 IU suggested a long-term infection, while an intensity below 2.9 IU indicated a recent infection.

This dual approach of visual and reader-based interpretation provided a comprehensive evaluation of HIV infection status in the specimens tested.

### 2.4. Data Analysis and Statistical Methods

#### 2.4.1. False Recent Rates (FRRs)

FRRs were calculated for both the LAg-Avidity EIA and the ARRA, including visual and strip reader interpretations. The FRR represents the proportion of long-term infections incorrectly classified as recent infections.

#### 2.4.2. Agreement Assessment

McNemar’s test was employed to evaluate the agreement between the categorical classifications of “recent” and “long-term” infections as determined by the LAg-Avidity EIA and ARRA. This test is particularly suited for paired data and assesses whether the observed agreement between two methods deviates significantly from what would be expected by chance. The use of McNemar’s test allows for a robust comparison of classification methods, particularly when evaluating diagnostic tools where outcomes are dichotomous (e.g., recent vs. long-term infections). The test results (*p* > 0.05) confirmed that there were no statistically significant differences in classification between the two methods.

#### 2.4.3. Correlation Analysis

Spearman’s rho was employed to assess the correlation between the Asanté™ HIV-1 Rapid Recency^®^ Assay (ARRA) reader-based results and the optical density (ODn) values from the LAg-Avidity Enzyme Immunoassay (LAg-Avidity EIA). Spearman’s rho is a non-parametric test that quantifies the strength and direction of the monotonic association between the two variables.

These analyses, which include both McNemar’s test for agreement and Spearman’s rho for correlation, ensure a comprehensive evaluation of the performance and agreement of the LAg-Avidity EIA and ARRA in classifying HIV infection status.

## 3. Results

### 3.1. Performance Evaluation of LAg-Avidity EIA and ARRA for Long-Term HIV Infection Specimens

A total of 634 serum or plasma specimens from individuals with long-term HIV infections (over one year and no antiretroviral therapy) were tested using the LAg-Avidity EIA, ARRA visual interpretation, and ARRA reader-based interpretation to classify them as recent or long-term infections. The LAg-Avidity EIA identified 2 specimens as recent infections and 632 as long-term. The ARRA visual interpretation classified all 634 specimens as long-term infections. The ARRA reader-based interpretation identified 2 specimens as recent infections and 632 as long-term, matching the LAg-Avidity EIA results (Table 1). Detailed results for the four discordant specimens are presented in Table 2.

The comparison is significant, as it underscores ARRA’s potential as a reliable point-of-care testing tool that delivers rapid results while maintaining strong concordance with the established gold standard, LAg-Avidity EIA. This is particularly important in resource-limited settings, where access to specialized laboratory infrastructure is constrained. By validating ARRA against LAg-Avidity EIA, this study provides evidence of its utility in improving the timeliness and accessibility of HIV surveillance programs, thereby supporting more effective epidemic monitoring and targeted intervention efforts.

### 3.2. False Recent Rate (FRR) Analysis

The false recent rate (FRR) measures the percentage of long-term infections misclassified as recent. In this analysis, both the LAg-Avidity EIA and ARRA reader-based interpretation demonstrated FRRs of 0.32%.

In contrast, the ARRA visual interpretation achieved perfect agreement with the LAg-Avidity EIA, yielding an FRR of 0.00%. This finding highlights the superior accuracy of ARRA visual interpretation in differentiating recent from long-term infections, with no false positives observed. The exceptional performance of the ARRA visual interpretation underscores its reliability and ease of use, particularly in diverse and resource-limited settings where access to sophisticated laboratory infrastructure is constrained. This near-perfect agreement supports the integration of ARRA into HIV surveillance programs, enabling real-time monitoring, improved targeting of public health interventions, and enhanced epidemic control efforts.

### 3.3. Agreement Between LAg-Avidity EIA and ARRA

McNemar’s test was utilized to evaluate the agreement between the categorical classifications of “recent” and “long-term” infections as determined by the LAg-Avidity EIA and ARRA, with comparative results summarized in Table 3. The visual interpretation of ARRA demonstrated a high level of agreement with LAg-Avidity EIA results (McNemar’s test, *p* = 0.500). Notably, no specimens were classified as recent infections using this method. The ARRA reader-based interpretation showed strong consistency with the LAg-Avidity EIA (McNemar’s test, *p* = 1.000), accurately identifying the same two specimens as recent infections. The test results (*p* > 0.05) indicated no statistically significant differences in classification between the two methods, further validating ARRA’s reliability and utility as a point-of-care testing tool.

### 3.4. Performance of ARRA Reader Versus LAg-Avidity EIA for Recent HIV Infection Detection

Spearman’s rank correlation coefficient (Spearman’s rho) was used to assess the relationship between the results obtained from the Asanté™ HIV-1 Rapid Recency^®^ Assay (ARRA) reader and the optical density (ODn) values derived from the HIV-1 Limiting Antigen (LAg)-Avidity Enzyme Immunoassay (LAg-Avidity EIA).

**Figure 1:**
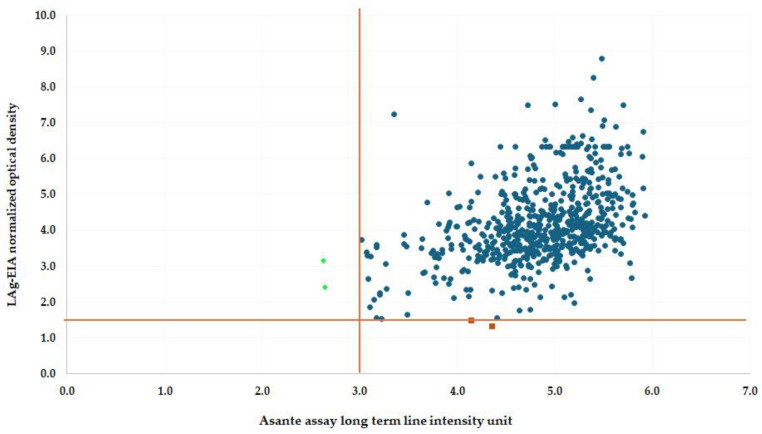
Correlation between the recency component of ARRA (Asanté reader-based) and LAg-Avidity EIA. The Spearman’s rho = 0.434. The figure illustrates the correlation between the recency classification results obtained from the ARRA (Asanté reader-based) and the LAg-Avidity EIA. Blue dots represent concordant results classified as “long-term” infections by both assays. Red dots indicate discordant results classified as “recent” by the LAg-Avidity EIA but “long-term” by the ARRA reader. Green dots represent discordant results classified as “recent” by the ARRA reader but “long-term” by the LAg-Avidity EIA.

A total of 634 specimens were tested using both the LAg-Avidity EIA and the ARRA reader. Spearman’s rho was calculated to evaluate the overall correlation between the two assays. The Spearman’s rho value was 0.434 (N = 634) (Figure 1), indicating a moderate positive correlation. This non-parametric analysis highlights the monotonic relationship between the assays, demonstrating that ARRA can approximate the trends observed with the established LAg-Avidity EIA.

While the results suggest a relationship between the two methods, differences in their ability to detect recent HIV infections were observed. These discrepancies underscore the importance of further investigation into the complementary roles of ARRA and LAg-Avidity EIA in HIV surveillance, particularly in diverse epidemiological settings.

### 3.5. Evaluation of ARRA Performance in High-Risk Populations with Unknown Infection Periods

The ARRA test kit was evaluated using HIV samples from individuals with unknown periods of infection, including pregnant women, female sex workers, and men who have sex with men (MSM), representing high-risk groups for HIV infection. The samples were initially tested using the LAg-Avidity EIA technique, and only those classified as long-term infections were subsequently analyzed with the ARRA test kit. Among these, the ARRA results identified 1 case from a pregnant woman as recent and 223 cases as long-term, yielding an inconsistency rate of 0.45% (Table 4).

## 4. Discussion

The UNAIDS 2024 report underscores the importance of targeted interventions in addressing the global HIV epidemic. Approximately 70% of new infections occur among key populations, including sex workers, men who have sex with men (MSM), transgender individuals, people who inject drugs, and their partners [1]. In Thailand, where HIV prevalence among transgender women (29.7%) and MSM (16.7%) is significantly high, these tools support the urgent need for tailored interventions [12]. This emphasizes the importance of accessible tools like ARRA to monitor and address transmission dynamics in these groups.

This study evaluated the performance of two assays, the LAg-Avidity EIA and ARRA, in distinguishing recent and long-term HIV infections using 634 serum or plasma specimens from individuals with long-term HIV diagnoses in Thailand. Both assays demonstrated high reliability, with comparable false recent rates (FRR) of 0.32%, confirming their potential utility in HIV incidence surveillance and public health interventions. While the LAg-Avidity EIA is considered the gold standard, providing critical data for HIV incidence estimation and transmission monitoring [4,9,15], its reliance on specialized laboratories, skilled personnel, and extended turnaround times limits its feasibility for point-of-care (POC) use. This highlights the need for accurate, adaptable alternatives for diverse healthcare settings [16]. In contrast to the LAg-Avidity EIA, the ARRA offers rapid, point-of-care testing with minimal infrastructure requirements, making it particularly suited for use in resource-limited settings. In this study, ARRA demonstrated strong agreement with the LAg-Avidity EIA, particularly when interpreted using the reader-based method. These findings align with previous studies conducted in Malawi and Rwanda, where ARRA effectively identified recent infections in high-risk populations and showed high concordance with the LAg-Avidity EIA [8,11,13].

One of the key advantages of ARRA is its ability to deliver results within minutes, significantly reducing turnaround times. The perfect agreement (FRR = 0.00%) achieved by ARRA’s visual interpretation with the LAg-Avidity EIA underscores its exceptional accuracy in distinguishing recent from long-term infections. This level of reliability and simplicity highlights ARRA’s potential as a dependable and efficient testing method. Additionally, ARRA has proven effective in identifying transmission clusters, enabling targeted interventions, and informing public health strategies in high-prevalence regions. These attributes establish ARRA as a valuable tool in advancing HIV epidemic control, particularly in underserved or hard-to-reach populations [17,18].

The ARRA test kit was also evaluated in high-risk populations with unknown infection periods, including pregnant women, female service workers, and MSM. Among 224 samples classified as long-term infections by the LAg-Avidity EIA, ARRA misclassified one case as a recent infection, resulting in an inconsistency rate of 0.45%. This low rate highlights the overall reliability of ARRA for detecting long-term infections compared to the LAg-Avidity EIA. These findings support the integration of ARRA as a POC tool for monitoring HIV recency and enhancing the efficiency of surveillance programs in resource-limited settings [16]. Spearman’s rho was calculated to assess the correlation between the LAg-Avidity EIA and ARRA reader results in long-term infections. The moderate correlation (Spearman’s rho = 0.434) suggests that, while ARRA aligns with the trends observed in the LAg-Avidity EIA, certain variations exist that warrant further exploration. Such systems could enhance the efficiency of surveillance programs and improve linkage to care, particularly in regions like sub-Saharan Africa, where approximately 84% of individuals are aware of their HIV status [19].

Future studies should broaden the scope of samples to include individuals with confirmed recent infections and incorporate CD4 count and viral load testing into the analyses. The implementation of a recent infection testing algorithm (RITA) in Thailand in 2022 presents an opportunity to enhance classification accuracy by integrating serological and virological markers, establishing a more robust framework for recency testing [16,20]. Additionally, longitudinal studies are recommended to assess the long-term performance of ARRA in real-world settings, with detailed sensitivity and specificity assessments across different populations. Including individuals at various stages of HIV infection, particularly those with confirmed recent infections, would enhance the research. Furthermore, the emergence of new HIV strains or mutations could impact the performance of ARRA and the LAg-Avidity EIA. Therefore, continuous evaluation against evolving strains is essential to maintain diagnostic accuracy and ensure these tools remain effective in diverse and dynamic epidemiological contexts.

## 5. Conclusions

Both the LAg-Avidity EIA and ARRA demonstrated strong performance in identifying long-term HIV infections. The ARRA, with its rapid and accessible design, presents a viable alternative for point-of-care testing in resource-limited settings. Integrating ARRA into routine HIV surveillance systems has the potential to significantly enhance public health efforts, particularly in regions with high HIV prevalence, such as Thailand. Its capability to generate real-time data on transmission dynamics and inform targeted interventions underscores its critical role in advancing strategies for HIV prevention and epidemic control.

## Figures and Tables

**Table 1:** Classification of HIV infection status by LAg-Avidity EIA, ARRA visual, and ARRA reader-based interpretation.

Assay	Recent Infections	Long-Term Infections	Total (N = 634)	False Recent Rate (FRR)
LAg-Avidity EIA	2 *	632	634	0.32%
ARRA Visual	0	634	634	0.00%
ARRA Reader-Based	2 *	632	634	0.32%

Notes: False recent rate (FRR): calculated as the percentage of long-term infections incorrectly identified as recent infections. N = 634: total number of specimens analyzed from individuals with long-term HIV infections. * The discordant classification of “recent” in the LAg-Avidity EIA and ARRA reader-based method.

**Table 2:** Detailed results for specimens with discordant classifications.

Specimen ID	LAg-Avidity EIA Classification (ODn)	ARRA Visual Interpretation	ARRA Reader-Based Interpretation (PVL, LTL Intensity)	Comments
80-8001-027	Long-term Infection (3.163)	Long-term Infection	* Recent Infection (5.19, 2.63)	Misclassified by ARRA reader
90-9010-143	Long-term Infection (2.419)	Long-term Infection	* Recent Infection (4.17, 2.64)	Misclassified by ARRA reader
77-7707-024	* Recent Infection (1.326)	Long-term Infection	Long-term Infection (5.91, 4.35)	Discrepancy between assays
77-7707-008	* Recent Infection (1.492)	Long-term Infection	Long-term Infection (5.59, 4.14)	Discrepancy between assays

Note: LAg-Avidity EIA Classification: Specimens with an ODn > 1.5 were classified as long-term infections, while those with an ODn ≤ 1.5 were classified as recent infections. Strip Reader Interpretation: PVL intensity > 2.8 IU indicates an HIV-positive result. LTL intensity > 2.9 IU suggests a long-term infection, while an intensity ≤ 2.9 IU indicates a recent infection. * = the discordant classification of “recent” in the LAg-Avidity EIA and ARRA reader-based method.

**Table 3:** Agreement between LAg-Avidity EIA and ARRA visual and reader-based interpretations (N = 634).

Tests	LAg-Avidity EIA (Recent)	LAg-Avidity EIA (Long-Term)	Total	Mcnemar’s Test *p* Value
ARRA Visual	0	634	634	0.500
ARRA Reader-Based	2 *	632	634	1.000

Notes: A *p*-value > 0.05 indicates no significant difference in classification between the two methods. * = The discordant classification of “recent” in the ARRA reader-based method.

**Table 4:** Performance of ARRA in long-term HIV infections among high-risk groups.

Group	Total Samples Tested	LAg-Avidity EIA Long-Term Results	ARRA Long-Term Results	ARRA Recent Results	Inconsistency Rate (%)
Pregnant women	130	130	129	1	0.77
Female sex workers	86	86	86	-	0.00
MSM	8	8	8	-	0.00
**Total**	224	224	223	1	0.45

Notes: MSM: men who have sex with men.

## Data Availability

Data are unavailable due to privacy.
